# Comprehensive Symptom Prediction in Inpatients With Acute Psychiatric Disorders Using Wearable-Based Deep Learning Models: Development and Validation Study

**DOI:** 10.2196/65994

**Published:** 2024-11-13

**Authors:** Minseok Hong, Ri-Ra Kang, Jeong Hun Yang, Sang Jin Rhee, Hyunju Lee, Yong-gyom Kim, KangYoon Lee, HongGi Kim, Yu Sang Lee, Tak Youn, Se Hyun Kim, Yong Min Ahn

**Affiliations:** 1 Department of Neuropsychiatry Seoul National University Hospital Seoul Republic of Korea; 2 Department of Psychiatry Seoul National University College of Medicine Seoul Republic of Korea; 3 Department of IT Convergence Engineering Gachon University Seongnam-si Republic of Korea; 4 Department of Psychiatry Chungnam National University Sejong Hospital Sejong Republic of Korea; 5 Department of Computer Engineering Gachon University Seongnam-si Republic of Korea; 6 Healthconnect Co. Ltd. Seoul Republic of Korea; 7 Department of Psychiatry Yong-In Mental Hospital Yongin-si Republic of Korea; 8 Department of Psychiatry and Electroconvulsive Therapy Center Dongguk University International Hospital Goyang-si Republic of Korea; 9 Institute of Buddhism and Medicine Dongguk University Seoul Republic of Korea; 10 Institute of Human Behavioral Medicine Seoul National University Medical Research Center Seoul Republic of Korea

**Keywords:** digital phenotype, mental health monitoring, smart hospital, clinical decision support system, multitask learning, wearable sensor, local validation, mental health facility, deep learning

## Abstract

**Background:**

Assessing the complex and multifaceted symptoms of patients with acute psychiatric disorders proves to be significantly challenging for clinicians. Moreover, the staff in acute psychiatric wards face high work intensity and risk of burnout, yet research on the introduction of digital technologies in this field remains limited. The combination of continuous and objective wearable sensor data acquired from patients with deep learning techniques holds the potential to overcome the limitations of traditional psychiatric assessments and support clinical decision-making.

**Objective:**

This study aimed to develop and validate wearable-based deep learning models to comprehensively predict patient symptoms across various acute psychiatric wards in South Korea.

**Methods:**

Participants diagnosed with schizophrenia and mood disorders were recruited from 4 wards across 3 hospitals and prospectively observed using wrist-worn wearable devices during their admission period. Trained raters conducted periodic clinical assessments using the Brief Psychiatric Rating Scale, Hamilton Anxiety Rating Scale, Montgomery-Asberg Depression Rating Scale, and Young Mania Rating Scale. Wearable devices collected patients’ heart rate, accelerometer, and location data. Deep learning models were developed to predict psychiatric symptoms using 2 distinct approaches: single symptoms individually (Single) and multiple symptoms simultaneously via multitask learning (Multi). These models further addressed 2 problems: within-subject relative changes (Deterioration) and between-subject absolute severity (Score). Four configurations were consequently developed for each scale: Single-Deterioration, Single-Score, Multi-Deterioration, and Multi-Score. Data of participants recruited before May 1, 2024, underwent cross-validation, and the resulting fine-tuned models were then externally validated using data from the remaining participants.

**Results:**

Of the 244 enrolled participants, 191 (78.3%; 3954 person-days) were included in the final analysis after applying the exclusion criteria. The demographic and clinical characteristics of participants, as well as the distribution of sensor data, showed considerable variations across wards and hospitals. Data of 139 participants were used for cross-validation, while data of 52 participants were used for external validation. The Single-Deterioration and Multi-Deterioration models achieved similar overall accuracy values of 0.75 in cross-validation and 0.73 in external validation. The Single-Score and Multi-Score models attained overall *R*² values of 0.78 and 0.83 in cross-validation and 0.66 and 0.74 in external validation, respectively, with the Multi-Score model demonstrating superior performance.

**Conclusions:**

Deep learning models based on wearable sensor data effectively classified symptom deterioration and predicted symptom severity in participants in acute psychiatric wards. Despite lower computational costs, Multi models demonstrated equivalent or superior performance to Single models, suggesting that multitask learning is a promising approach for comprehensive symptom prediction. However, significant variations were observed across wards, which present a key challenge for developing clinical decision support systems in acute psychiatric wards. Future studies may benefit from recurring local validation or federated learning to address generalizability issues.

## Introduction

Assessing psychiatric symptoms of patients with acute psychiatric disorders remains challenging [1-3]. Patients with psychosis, mania, and severe depression who require hospitalization often struggle to accurately report their symptoms. Poor insight, a short attention span, impaired cognition, paranoia, and severe avolition can result in underreporting, minimization, and impairment of the verbalization process [3,4]. Additionally, the inherent subjectivity in assessing psychopathology presents reliability concerns and requires experienced professionals [5,6]. Even well-staffed and equipped wards cannot provide continuous patient observation by trained medical personnel.

Recent advancements in wearable sensor technology and artificial intelligence (AI) have enabled the collection and analysis of vast amounts of data [7-9]. These data encompass characteristics related to human behavior, cognition, and mood associated with mental disorders [10] and are often referred to as digital phenotyping [11-13]. The continuous and objective measurement capabilities of sensor data can overcome the limitations of traditional psychiatric assessments [14-16]. Numerous studies have reported that sensor data from wearables and mobile phones can significantly predict health outcomes, such as disease risk and mortality, by unobtrusively monitoring sleep [17-20], circadian rhythms [21-23], and physical activity [24,25].

Using sensor data in acute psychiatric wards is expected to produce significant advantages. Acute psychiatric wards must always be prepared for the risks of aggression and self-harm, resulting in high staffing demands [26,27]. Health care workers in these settings are repeatedly exposed to such patient behaviors, introducing a significant risk of burnout [28,29]. Additionally, human rights standards and the legislation required for the care of psychiatric inpatients are progressing globally [30]. Consequently, digital technology adoption remains a pressing issue in addressing these challenges in psychiatric wards [31]. Furthermore, psychiatric wards provide a highly controlled environment, enabling accurate and continuous collection of wearable sensor data and detailed clinical information. This creates suitable conditions for digital phenotyping research [32].

Despite these expected advantages, only a few studies have presented AI models to support medical personnel in the context of acute psychiatric wards. To the best of our knowledge, the task of symptom prediction has not yet been addressed. Psychiatric wards exhibit a wide range of architectural, staffing, and organizational variations [33-35], likely contributing to limited research in these contexts. Furthermore, psychiatric inpatients have diverse diagnoses and a wide symptom range [36,37]. Predicting comprehensive symptoms across multi-institutional pandiagnostic groups is necessary to provide a robust foundation for AI-assisted clinical decision support systems (AI-CDSSs) [38].

Therefore, we aimed to develop and validate a comprehensive symptom prediction model for patients in acute psychiatric wards using sensor data obtained using wearable devices and deep learning–based prediction models. This prospective, longitudinal, observational study was conducted across multiple institutions, representing various regions and hospital types. We constructed and evaluated models for predicting single symptoms individually and multiple symptoms simultaneously, as well as for predicting relative changes within subjects and absolute severity between subjects. Moreover, we explored the future directions and challenges of AI-CDSSs in psychiatric ward settings.

## Methods

### Overview

The study was presented according to the Guidelines for Developing and Reporting Machine Learning Predictive Models in Biomedical Research [39], as well as the TRIPOD+AI guideline (version February 7, 2024) [40].

### Ethical Considerations

This study was conducted in accordance with the Declaration of Helsinki [41]. The study protocol was reviewed and approved by the Institutional Review Board of the Seoul National University Hospital (2210-073-1368). All participants provided informed consent. Participants received compensation of KRW 100,000 (approximately US $70) for each week of participation. Research data were stored on servers within each hospital, separate from personally identifiable information except for study identification numbers. Access to and analysis of the data were restricted to preapproved researchers only. No identifiable images or personal information of participants are included in this manuscript or any supplementary materials.

### Participants

The Sensor Application for Early Response in Closed Wards (SAFER) project aims to build a generalizable wearable-based AI-CDSS for acute psychiatric wards. This project is ongoing until 2024, and interim data were used in this study.

Four wards from 3 hospitals representing diverse regions and hospital types participated in the SAFER study: Seoul National University Hospital, a tertiary general hospital in the capital city with a single participating ward; Yongin Mental Hospital, a suburban psychiatric hospital with 2 wards, one for each sex; and Dongguk University Ilsan Hospital, a general hospital in a newly developed city with one participating ward.

Inpatients aged 13 years or older, diagnosed with a mood disorder (major depressive or bipolar disorder) or schizophrenia spectrum disorder, were recruited from each ward. For vulnerable participants, including minors and individuals whose symptoms might impair their ability to understand and consent, additional consent was obtained from their legal guardians. Exclusion criteria included dementia, intellectual disability, organic brain disorders, and any physical condition causing difficulty in data collection (eg, wrists too thin owing to low body weight of participants with anorexia nervosa). However, other coexisting diagnoses were included to ensure a pandiagnostic study population. As one of the analysis targets was a comparison with previous scores of the same individual, the analysis included only participants who had undergone at least 2 assessments, including the baseline assessment.

### Measurements

Each participant was provided with a wrist-worn device called URBAN HR (Partron Co, Ltd). Participants were instructed to wear the device continuously, except when showering or leaving the ward, although they could remove it freely if desired. The wearable devices collected data on heart rate, 3-axis acceleration (which measures acceleration along 3 perpendicular axes in space), and location. Using in-device algorithms, these metrics were used to calculate calories burned, steps walked, distance moved, and sleep index. Among these, the sleep index is used to analyze participants’ sleep and is calculated based on heart rate and acceleration data using algorithms provided by the manufacturer. This sleep index quantifies the cumulative daily sleep duration, converting it into a score ranging from 30 (2.25 h or less) to 100 (7.5 h or more). The generated data were transmitted in real time to the server at each hospital via Bluetooth gateways installed in each patient’s room and common areas.

The participants were assessed weekly for symptoms by raters using clinician-rated scales. Additional assessments were conducted following injection, seclusion, and restraint interventions: the Brief Psychiatric Rating Scale (BPRS) was used to evaluate psychotic and general psychiatric symptoms, the Hamilton Anxiety Scale (HAM-A) was used to assess anxiety, the Montgomery-Asberg Depression Rating Scale (MADRS) was used to evaluate depressive symptoms, and the Young Mania Rating Scale (YMRS) was used to measure manic symptoms. The raters were registered nurses with a minimum of 51 months and an average of 11 years of clinical experience. All raters completed 40 hours of specialized training for the study, and their ratings were periodically reviewed at research conferences with psychiatrists.

### Feature Engineering

A 1-hour sliding window was used to synthesize time-series values from various sensors. The 3-axis accelerations were converted to a total acceleration, which is the magnitude of the summed acceleration. The first value, last value, mean, median, maximum, minimum, SD, and number of unique values within the 1-hour sliding window were calculated for total acceleration and heart rate. Calories burned, sleep index, number of steps walked, and distance traveled were calculated as cumulative values over this window. Regarding location data, the variance of the coordinate values and most frequent semantic location (room, hallway, other, and no signal) were computed over the sliding window.

Location entropy quantifies the diversity and unpredictability of a participant’s location patterns, which have demonstrated predictive value for mental health correlates [42-44]. We computed entropy values over 24-hour and 8-hour periods, delineated by midnight, 8 AM, and 4 PM. This approach was chosen as meaningful location entropy assessment requires longer periods than the 1-hour sliding window.

Missing values in the sensor data were imputed using the *softImpute* method [45]. The participant was excluded if missing values exceeded 50% of their total timespan. Sensor data from the 4 weeks preceding each symptom assessment were used in the model. In cases where available data preceding the assessment were less than 4 weeks, zero-padding was applied to extend the data to the required length [46]. The size of the input layer was 672 hours with 31 features (672 × 31) including 2 nonsensor features (age and sex). t-distributed Stochastic Neighbor Embedding (t-SNE) was used to visualize the distribution of these 31 features by ward.

### Prediction Model and Performance Evaluation

**Figure 1:**
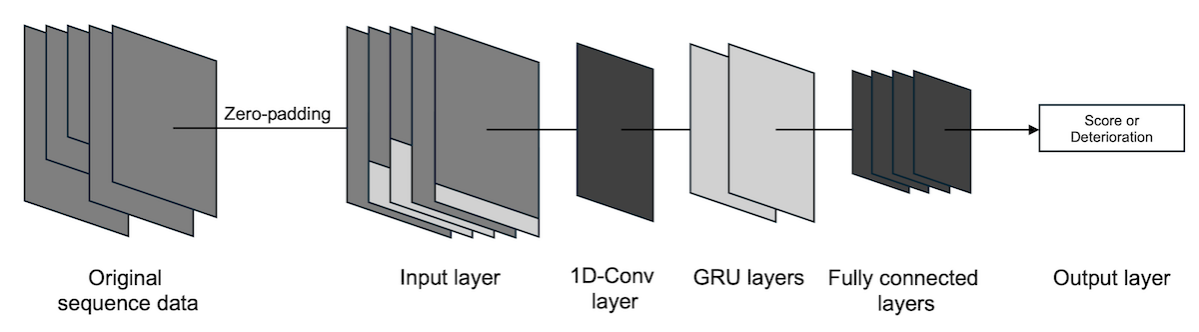
The architecture of deep learning models for predicting comprehensive symptoms in inpatients with acute psychiatric disorders. Original sequence data were adjusted to match the required length by removing data older than 4 weeks; shorter data were zero-padded to form the input layer. 1D-Conv, GRU, and fully connected layers were used sequentially. The model’s performance was compared based on various output layer configurations. 1D-Conv: 1D convolutional; GRU: gated recurrent unit.

A model designed to predict symptoms encompassing the BPRS, HAM-A, MADRS, and YMRS was constructed using a deep learning architecture that incorporates 1D convolutional layers and gated recurrent units (Figure 1). All models shared an identical structure, except for the terminal fully connected layer and output layer.

These models were differentiated based on the configuration of the output layer according to 2 criteria. First, the models were classified based on whether they predicted a single scale at a time (Single) or predicted all scales simultaneously (Multi). Multi models used a multitask learning (MTL) approach [47]. Second, the models were distinguished based on whether they classified instances where the symptom scale score increased from the previous assessment within an individual (Deterioration) or predicted the absolute severity score of the symptoms (Score). Deterioration models corresponded to a within-subject design, while Score models aligned with a between-subject design [48]. Consequently, 4 types of models were developed for each scale by intersecting these 2 criteria: Single-Deterioration, Single-Score, Multi-Deterioration, and Multi-Score.

Participants recruited prior to May 1, 2024, were allocated to the internal cross-validation set, while those recruited on or after May 1, 2024, were assigned to the external validation set. The internal cross-validation set underwent a 5-fold cross-validation. Hyperparameters were optimized using the random search method [49]. The final model was evaluated on the external validation set. The performance of Deterioration models was evaluated using accuracy, area under the curve (AUC), and receiver operating characteristic (ROC) curve. In the cross-validation, the ROC curve was generated by vertically pooling the curves from each fold. The performance of Score models was assessed using *R*^2^ and normalized root mean squared error (NRMSE), with NRMSE chosen to account for varying score ranges across scales. Permutation feature importance was measured for features in the external validation set. The deep learning model was built using *PyTorch* (version 2.2) [50], and model performance evaluation and t-SNE were performed using *scikit-learn* (version 1.3) [51].

## Results

Overall, 244 participants were enrolled in the SAFER study between May 26, 2023, and August 5, 2024. Sixteen participants were excluded based on the exclusion criteria or consent withdrawal after initially agreeing to participate. Additionally, 37 participants were excluded from the analysis because they lacked at least 1 assessment or had > 50% missing data from wearable sensors. Ultimately, data from 191 participants, encompassing 3954 person-days of observation, were analyzed. Participants were observed for a mean of 20.7 (SD 17.5) days, with 4.3 (SD 2.9) assessments per participant. In the final sample, 57.6% (110/191) were female, and the most frequent diagnosis was a mood disorder with a current depressive episode (93/191, 48.7%). For validation purposes, 139 participants were assigned to the cross-validation set and 52 participants were assigned to the external validation set.

Significant differences were observed in sex, age, and household income across hospitals (all *P*<.001). The first psychiatric admission (*P*=.17) did not show any significant differences nor did BPRS (*P*=.45) or HAM-A (*P*=.27) scores, suggesting general clinical severity was similar. However, significant differences were observed in the number of assessments (*P*=.02), days of observation (*P*=.007), diagnostic groups (*P*=.005), and MADRS (*P*=.002) and YMRS (*P*<.001) scores across the hospitals (Table 1).

**Table 1:** Basic characteristics and statistical comparison of participants by hospital. The Kruskal-Wallis test was performed for continuous variables, and the chi-square test was performed for categorical variables.

		Total (N=191)	Hospital 1^a^ (n=73)	Hospital 2^b^ (n=88)	Hospital 3^c^ (n=30)	Statistics					*P* value	
						Chi-square (*df*)		Kruskal-Wallis test (*H*)				
**Sex, n (%)**							19.6 (2)		—^d^			<.001
	Male	81 (42.4)	17 (23.3)	51 (58.0)	13 (43.3)							
	Female	110 (57.6)	56 (76.7)	37 (42.0)	17 (56.7)							
**Age (years)**							—		21.5			<.001
	Mean (SD)	32.6 (13.8)	27.2 (10.7)	35.0 (13.5)	38.7 (17.1)							
	Median (IQR)	27 (21-40)	24 (19-29)	30 (22-45)	32 (24-52)							
**Diagnostic group, n (%)**							14.8 (4)		—			.005
	Schizophrenia spectrum	72 (37.7)	19 (26.0)	34 (38.6)	19 (63.3)							
	Mood-depressive^e^	93 (48.7)	44 (60.3)	39 (44.3)	10 (33.3)							
	Mood-manic^f^	26 (13.6)	10 (13.7)	15 (17.0)	1 (3.3)							
**Household income (KRW 5 million=US $3600), n (%)**							24.1 (2)		—			<.001
	>KRW 5 million	78 (40.8)	32 (43.8)	23 (26.1)	23 (76.7)							
	≤KRW 5 million	113 (59.2)	41 (56.2)	65 (73.9)	7 (23.3)							
**Education level, n (%)**							8.5 (4)		—			.07
	≥College	48 (25.1)	16 (21.9)	25 (28.4)	7 (23.3)							
	High school	118 (61.8)	41 (56.2)	57 (64.8)	20 (66.7)							
	≤Middle school	25 (13.1)	16 (21.9)	6 (6.8)	3 (10.0)							
**Number of assessments**							—		7.7			.02
	Mean (SD)	4.3 (2.9)	4.1 (2.8)	4.6 (3.1)	3.5 (2.8)							
**Observational days**							—		9.9			.007
	Mean (SD)	20.7 (17.5)	19.4 (14.5)	23.4 (19.3)	15.8 (17.5)							
**Baseline symptom score, mean (SD)**												
	BPRS^g^	21.5 (9.1)	22.8 (10.9)	20.6 (8.1)	21.2 (6.7)	—		1.6		.45		
	HAM-A^h^	11.2 (6.4)	10.2 (4.6)	12.0 (8.2)	11.5 (3.3)	—		2.6		.27		
	MADRS^i^	18.8 (10.9)	22.0 (9.7)	16.8 (12.4)	16.7 (6.2)	—		12.2		.002		
	YMRS^j^	11.1 (8.3)	9.8 (8.5)	10.7 (8.4)	15.5 (6.1)	—		17.5		<.001		
**First psychiatric admission, n (%)**							3.5 (2)		—			.17
	Yes	66 (34.6)	26 (35.6)	34 (38.6)	6 (20.0)							
	No	125 (65.4)	47 (64.4)	54 (61.4)	24 (80.0)							

^a^Hospital 1: Seoul National University Hospital.

^b^Hospital 2: Yongin Mental Hospital.

^c^Hospital 3: Dongguk University Ilsan Hospital.

^d^Not applicable.

^e^Mood-depressive: Mood disorder with current depressive episode.

^f^Mood-manic: Mood disorder with current manic episode.

^g^BPRS: Brief Psychiatric Rating Scale.

^h^HAM-A: Hamilton Anxiety Rating Scale.

^i^MADRS: Montgomery-Asberg Depression Rating Scale.

^j^YMRS: Young Mania Rating Scale.

The group-level mean for the scale scores were 15.9 (SD 9.5) for the BPRS, 8.8 (SD 6.0) for the HAM-A, 14.1 (SD 10.5) for the MADRS, and 8.1 (SD 7.1) for the YMRS, with no significant differences between the cross-validation and external validation sets (all *P*>.05; Table S1 in Multimedia Appendix 1). The deterioration of symptom cases, where the assessment score was increased to that of the previous assessment for an individual, were 22.9% (143/624) for the BPRS, 26.1% (163/624) for the HAM-A, 28.8% (180/624) for the MADRS, and 28.2% (176/624) for the YMRS; no significant differences were observed between the cross-validation and external validation sets (all *P*>.05; Table S2 in Multimedia Appendix 1).

**Figure 2:**
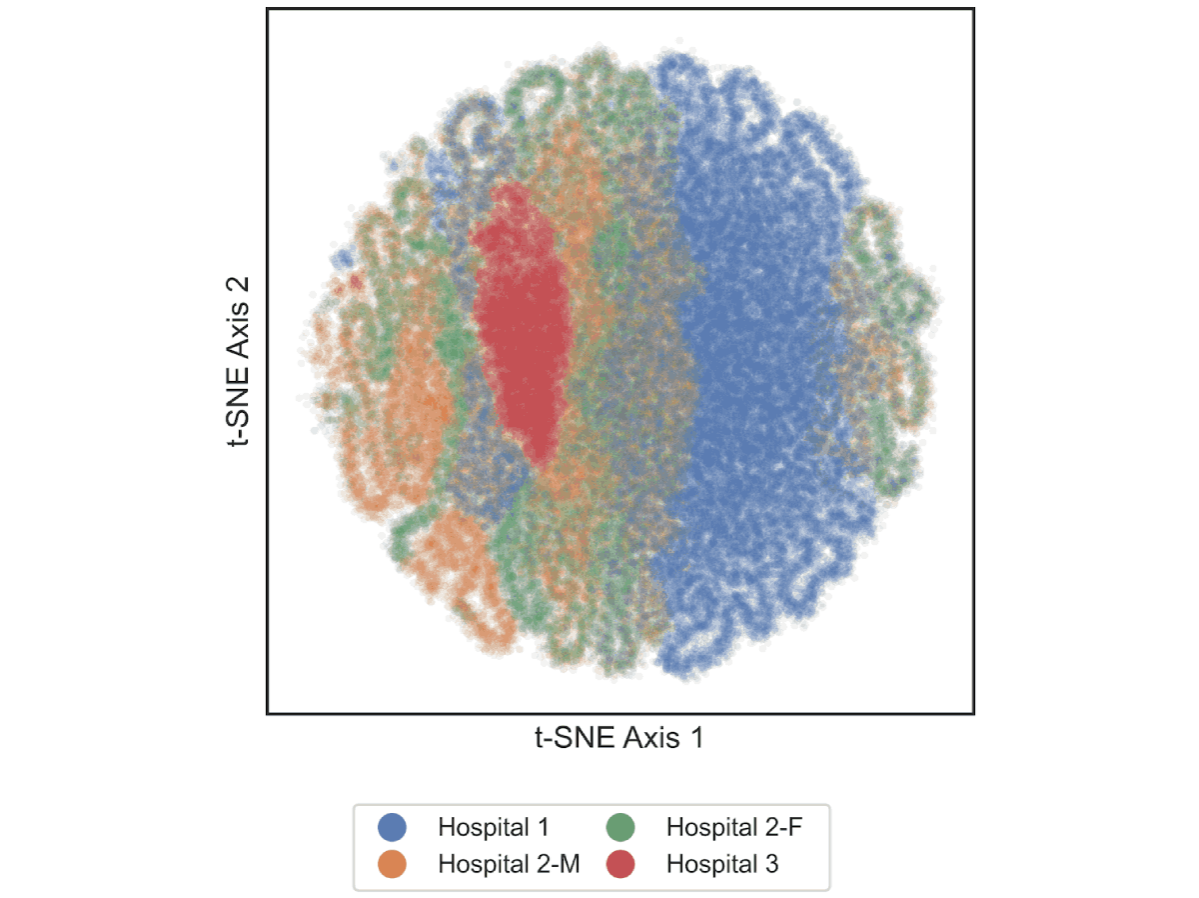
Visualization of the distribution of sensor data using t-SNE. Each point is color-coded to differentiate between hospital wards—hospital 1: Seoul National University Hospital; hospital 2: male (M) and female (F) wards in Yongin Mental Hospital; and hospital 3: Dongguk University Ilsan Hospital. t-SNE: t-distributed stochastic neighbor embedding.

The distribution of the multidimensional sensor data visualized using t-SNE revealed several clusters. Many clusters comprised data originating from 1 or 2 wards. Clusters with overlapping data from all wards were rare (Figure 2).

**Figure 3:**
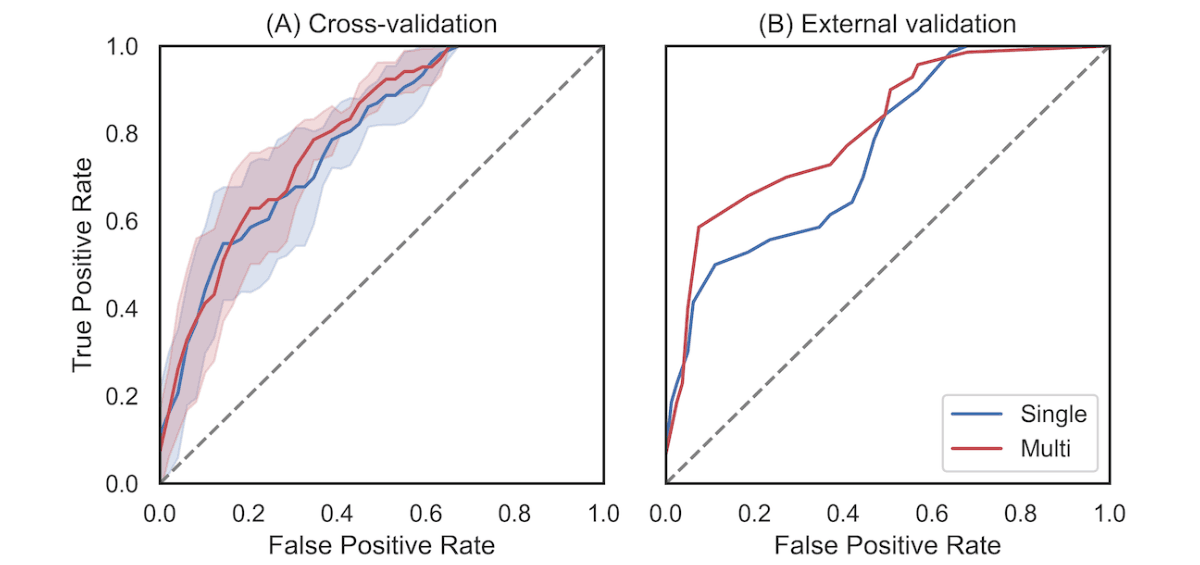
Receiver operating characteristic curve of the Deterioration models with respect to BPRS scores: (A) cross-validation and (B) external validation. The Deterioration models predict whether BPRS scores increased compared with the previous assessment. Colored areas in cross-validation represent the range of 1 SD. BPRS: Brief Psychiatric Rating Scale.

In the Deterioration models, the Single-Deterioration and Multi-Deterioration models showed similar overall performance. Cross-validation revealed that both models achieved an accuracy of 0.75, with an AUC of 0.74 and 0.76, respectively. External validation revealed an accuracy of 0.73 for both models, with an AUC of 0.71 and 0.74, respectively. Among the scales, the BPRS demonstrated the highest accuracy across all models and sets, with accuracy ranging from 0.75 to 0.79 and AUC from 0.76 to 0.82. The MADRS performed the least accurately in cross-validation and the the YMRS performed the least accurately in external validation, regardless of model type (Table 2). The ROC curve for the BPRS in cross-validation showed no clear superiority between Single-Deterioration and Multi-Deterioration models within 1 SD. External validation indicated that the 2 models’ curves intersected at both ends of the false positive rate range, with the Multi-Deterioration model generally outperforming in the central portion (0.1 to 0.6; Figure 3). Similar patterns were observed for other scales (Figures S1-3 in Multimedia Appendix 1).

**Table 2:** Performance of the Deterioration models measured by accuracy and area under the curve (AUC). The Deterioration models predict whether scale scores increased compared with the previous assessment.

		Cross-validation, mean (SD)		External validation		
		Single	Multi	Single	Multi	
**Accuracy**						
	Overall	0.75 (0.02)	0.75 (0.03)	0.73		0.73
	BPRS^a^	0.79 (0.02)	0.79 (0.03)	0.79		0.75
	HAM-A^b^	0.76 (0.05)	0.75 (0.05)	0.76		0.75
	MADRS^c^	0.72 (0.03)	0.73 (0.05)	0.74		0.75
	YMRS^d^	0.73 (0.04)	0.74 (0.02)	0.65		0.69
**AUC**						
	Overall	0.74 (0.04)	0.76 (0.04)	0.71		0.74
	BPRS	0.79 (0.07)	0.80 (0.06)	0.76		0.82
	HAM-A	0.75 (0.06)	0.77 (0.05)	0.73		0.75
	MADRS	0.69 (0.03)	0.72 (0.05)	0.69		0.73
	YMRS	0.73 (0.05)	0.76 (0.04)	0.64		0.67

^a^BPRS: Brief Psychiatric Rating Scale.

^b^HAM-A: Hamilton Anxiety Rating Scale.

^c^MADRS: Montgomery-Asberg Depression Rating Scale.

^d^YMRS: Young Mania Rating Scale.

**Figure 4:**
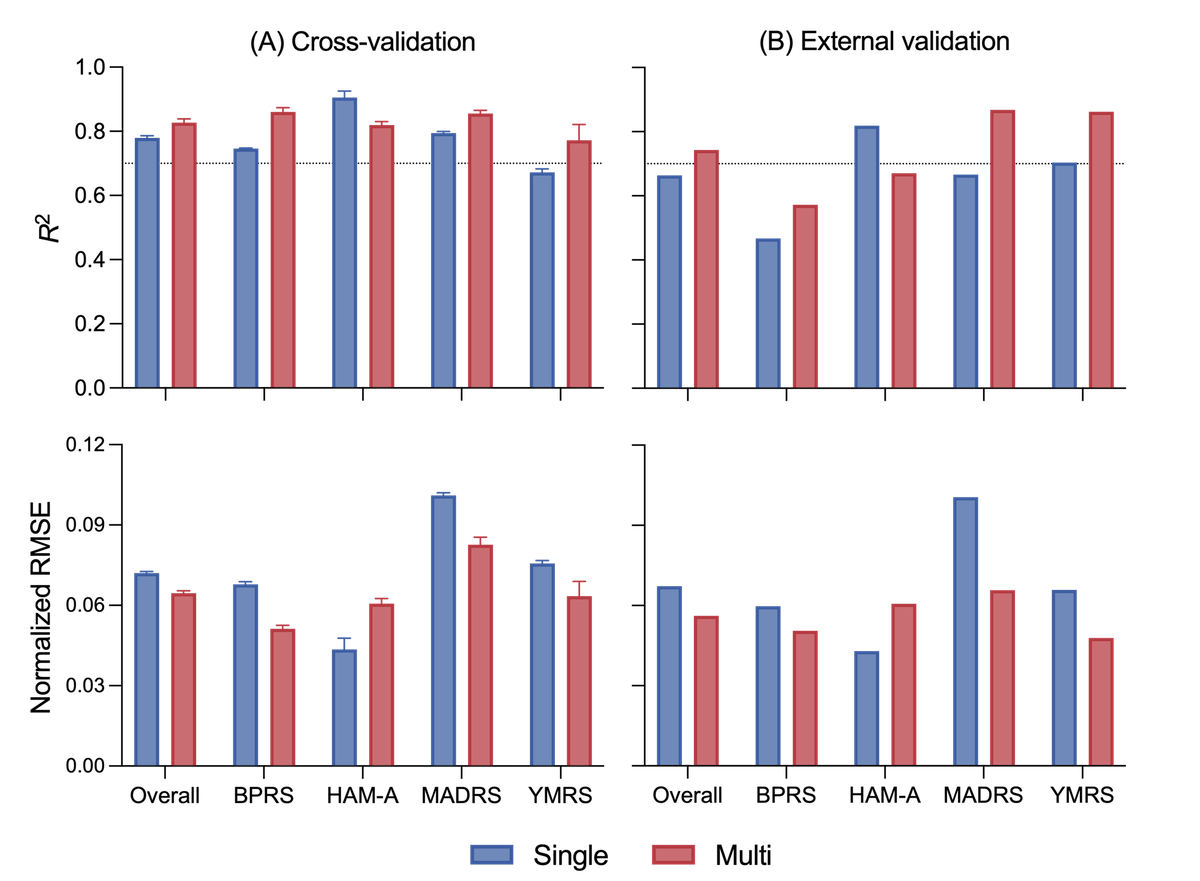
Performance of the Score model measured by R2 and NRMSE: (A) cross-validation and (B) external validation. The Score models predict scale scores; the error bar represents an interval of 1 SD. The dotted line indicates an R2 value of 0.7. BPRS: Brief Psychiatric Rating Scale; HAM-A: Hamilton Anxiety Rating Scale; MADRS: Montgomery-Asberg Depression Rating Scale; Multi: model predicting multiple symptoms simultaneously; NRMSE: normalized root mean squared error; Single: model predicting single symptoms individually; YMRS: Young Mania Rating Scale.

The Multi-Score model outperformed the Single-Score model in both *R*^2^ and NRMSE. In the cross-validation set, the Single-Score and Multi-Score models achieved *R*^2^ values of 0.78 and 0.83 and NRMSE values of 0.072 and 0.064, respectively. External validation showed that the Single-Score and Multi-Score models achieved *R*^2^ values of 0.66 and 0.74 and NRMSE values of 0.067 and 0.056, respectively. Among the scales, the BPRS, which was predicted well in the Deterioration models, demonstrated the lowest *R*^2^ values during external validation: 0.47 for Single-Score and 0.57 for Multi-Score. In contrast, the MADRS and YMRS exhibited *R*^2^ values exceeding 0.85 in the external validation of the Multi-Score model (Figure 4).

**Figure 5:**
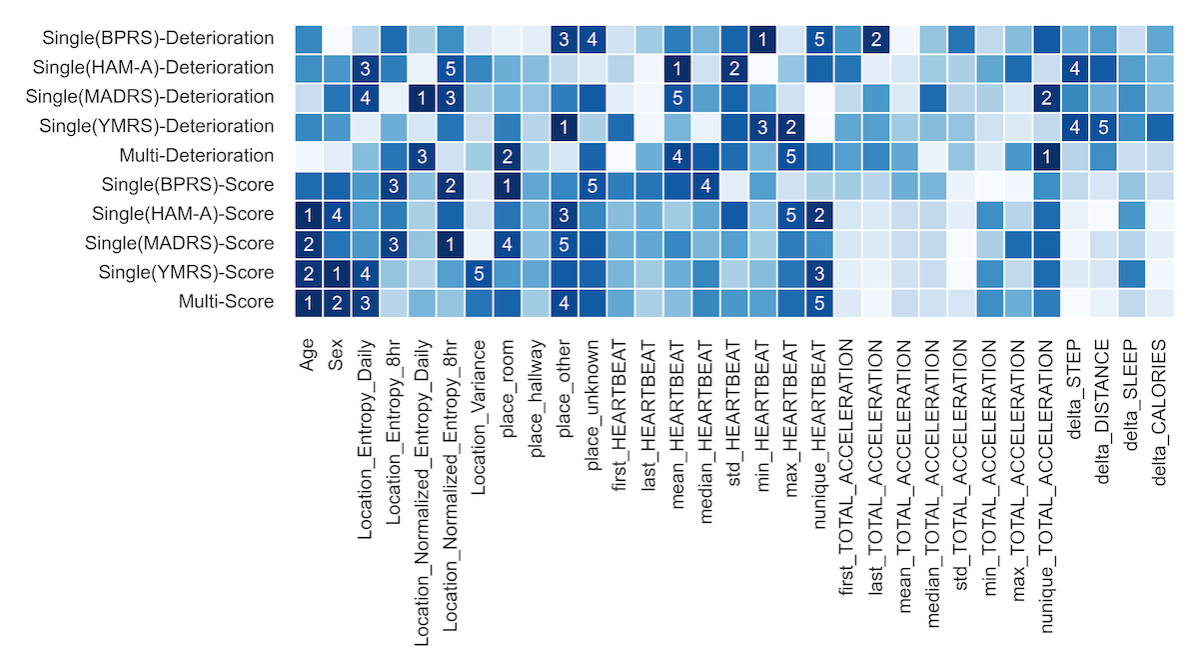
Permutation feature importance in external validation. The horizontal axis represents 31 individual features, and the vertical axis represents the prediction models. To ensure comparability, the importance values are converted to ranks, with the top 5 annotated. BPRS: Brief Psychiatric Rating Scale; HAM-A: Hamilton Anxiety Rating Scale; MADRS: Montgomery-Asberg Depression Rating Scale; Multi: model predicting multiple symptoms simultaneously; Single: model predicting single symptoms individually; YMRS: Young Mania Rating Scale.

The permutation feature importance analysis indicated that time spent in places other than rooms or hallways (*place_other*, 5 models), the number of unique heart rate values (*nunique_HEARTBEAT*, 4 models), and 2 location entropy indicators (*Location_Entropy_Daily* and *Location_Normalized_Entropy_8hr*, 4 models each) were among the top 5 important features in 4 or more models. Notably, no single feature consistently ranked in the top 5 across more than half of the models. Nonsensor features such as age and sex did not rank in the top 5 important features for the Deterioration models but were frequently included in the Score models (Figure 5).

## Discussion

### Principal Findings

We conducted a longitudinal, prospective, multicenter study of inpatients using wearable devices to develop and validate deep learning models for predicting comprehensive symptoms. To our knowledge, this is the first study to predict clinical rating scale scores of pandiagnostic cases in acute psychiatric wards using wearable devices and deep learning models. While direct benchmarks are unavailable owing to our inpatient setting, we can compare our results to those of similar studies. A meta-analysis of depression prediction studies using wearable devices reported a pooled mean accuracy ranging from 0.70 to 0.89 [52]. Similarly, a meta-analysis of anxiety prediction studies using wearable devices reported a 95% CI for pooled mean accuracy of 0.71 to 0.89 [53]. Our Single-Deterioration and Multi-Deterioration models for MADRS, HAM-A, and overall performance fall within these ranges, demonstrating comparable performance. The Single-Score and Multi-Score models showed overall *R*^2^ values of approximately 0.8 in cross-validation and 0.7 in external validation, indicating substantial explanatory power. In summary, our wearable-based deep learning models effectively predict comprehensive psychiatric symptoms.

### Single Versus Multi Models

Our Multi models used an MTL framework to simultaneously predict 4 different scale scores. While Single models might be expected to outperform Multi models, given that they require approximately 4 times the computational resources and parameters to learn and solve the same tasks [47], our findings suggest otherwise. The Single-Deterioration and Multi-Deterioration models, which predict changes from previous assessments for an individual, showed no material differences in both accuracy and AUC. Moreover, Multi-Deterioration models demonstrated a superior balance between sensitivity and specificity, as the AUC value and ROC curve indicated. Considering the class imbalance in symptom deterioration cases, this suggests that MTL-based models provide a more balanced predictive performance. Furthermore, the Single-Score and Multi-Score models, which predict absolute symptom severity, showed that the Multi-Score model demonstrated better explanatory power and lower errors in both cross-validation and external validation.

This study is not the first to attempt to predict mental health indicators using MTL-based models. Several studies have reported significant success using this approach with data from social media [54], electrocardiography [55], functional magnetic resonance imaging [56], and so on. Interestingly, while Harvey et al [57] argued that thousands of samples might be necessary for MTL to show benefits over single-task learning in predicting psychiatric diagnoses using functional magnetic resonance imaging, our study demonstrated these benefits with fewer samples. Although the comparability is limited due to the difference between cross-sectional brain imaging and longitudinal wearable sensor data, a notable distinction is that they predicted diagnoses, whereas we predicted symptoms.

Many contemporary psychopathologists agree that individual psychiatric symptoms form a complex network [58,59]. From this perspective, symptoms are not merely passive manifestations of specific diagnoses but active entities influencing other symptoms [60]. Thus, information learned in predicting one symptom can inform the prediction of others. In contrast, psychiatric diagnoses are defined through operational criteria of symptom constellations [61]. Consequently, learning shared latent information for multiple symptom prediction may be more feasible and may require fewer samples than those required for predicting multiple diagnoses. A similar study predicting symptoms in individuals with schizophrenia using smartphone passive data found benefits from MTL with only 61 samples [19]. This was likely possible because the prediction targets were individual symptoms rather than diagnoses. Although there are not yet many studies directly comparable with the present research, our study findings suggest that MTL is an effective framework for psychiatric symptom prediction tasks.

### Deterioration Versus Score Models

The Deterioration and Score models exhibit several notable differences. The purpose of the Deterioration models was to detect symptom changes within individuals compared with their previous state, whereas the Score models aimed to predict the absolute symptom severity between individuals. For feature importance, no shared patterns of important features were observed between the Deterioration and Score models predicting the same symptom scale. For instance, age and sex emerged as important in most Score models but not in Deterioration models. This is likely because predicting absolute symptom severity relies more on basic demographic information as crucial calibration data compared with predicting relative changes. Performance patterns during external validation also varied across scales. The BPRS demonstrated the best performance among all scales in both cross-validation and external validation for the Deterioration models, but the Score models showed a considerable *R*^2^ reduction in external validation. Conversely, the YMRS exhibited the most significant performance decline in external validation for the Deterioration models but not for the Score models. This indicates that the Deterioration and Score models, even when predicting the same symptom scale, differ in their required information and generalization challenges. This serves as another example demonstrating the distinction between within-subject and between-subject designs [62]. Future studies on predicting symptoms in acute psychiatric inpatients should consider treating relative change and absolute severity as distinct analyses.

### Challenges to Generalizability in AI-CDSSs for Acute Psychiatric Wards

The generalizability of models is a critical issue in developing AI models for predicting mental health indicators [63,64]. SAFER’s ultimate goal was to develop a predictive model that can serve as the foundation for an AI-CDSS in acute psychiatric wards with broad generalizability. In this study, external validation based on time point maintained considerable performance compared with internal cross-validation, demonstrating the model’s temporal robustness at least for the study period. However, recent studies have pointed out that external validation alone cannot fully guarantee reliability and generalizability in clinical prediction models [65]. In this study, challenges to generalizability remain in 2 aspects.

One challenge is interward variations. In this study, participant characteristics and sensor data distributions showed considerable differences across institutions. This was partly inevitable owing to the study’s aim to include diverse regions and hospital types. Nevertheless, the observed substantial interward variation suggests that applying an AI-CDSS based on this study to any other facility may encounter significant generalization difficulties. Studies on other clinical prediction models have noted that even models developed from large international multisite clinical trials often see their performance drop to chance levels when subjected to completely independent tests [66]. A potential solution to this issue is a method called recurring local validation or targeted validation [67,68]. This method involves validation and adjustment of prediction models to fit the specific settings, where they will be deployed. Future research should explore whether such ward-specific models could replace a single universal model.

The other challenge is the issue of selection bias [69]. In this study, we recruited pandiagnostic cases by minimizing the exclusion criteria based on clinical conditions and accepting various comorbidities. One reason for needing such an inclusive sample is that the end users of an AI-CDSS in acute psychiatric wards are primarily the ward’s medical staff [31]; thus, its usability can only be ensured if it can be applied with minimal restrictions to patients in the ward. However, participants in this study were recruited based on informed consent. Even with minimized exclusion criteria, patients with acute psychiatric symptoms may be subject to selection bias owing to differences in their ability to consent caused by their symptoms, making it difficult to recruit participants in a completely inclusive manner, like those with stroke or Alzheimer disease [70,71]. Therefore, in the future deployment of this model, validation based on data collected close to usual care, similar to the concept of pragmatic trials, will be necessary [72]. Waivers of the requirement for informed consent could be helpful in this case, but they require various ethical and regulatory considerations [73]. One of these is that the collected sensor data contains sensitive personal information, which makes it difficult to analyze through data centralization in one place. A promising solution to this could be engineering techniques such as federated learning, which allows for learning and validation without exporting raw data outside the institution [74].

### Limitations

First, although external validation was performed, it was conducted by dividing participants based on recruitment periods rather than by institutions. As confirmed in this study, acute psychiatric facilities varied significantly from one another, resulting in inherent generalizability issues. Thus, while the significance of the external validation is somewhat limited, we discussed various challenges and potential solutions to improve the model’s generalizability. Second, this study was conducted in acute psychiatric wards within the context of South Korea’s specific ethnic, sociocultural, legislative, and reimbursement systems. These contextual factors surrounding mental health facilities differ considerably between countries [30,75]. Therefore, developing a comprehensive symptom prediction model based on this study in other countries would require substantial consideration of these factors. Third, the average duration of the study was <3 weeks, which is relatively short. Therefore, patients with shorter treatment durations may have been overrepresented, while relatively few participants provided data over a sufficiently long observation period. Fourth, the sample size was insufficient to fully cover the objectives of this study, which aimed at a diverse population across various settings. In particular, the number of participants older than 40 years or with low educational attainment was small, and the usefulness of our clinical prediction model may be reduced for participants with these demographic characteristics. Fifth, different raters were responsible for clinical assessments at each hospital. Efforts were made to improve measurement reliability through specialized training courses and periodic research conferences; however, interrater errors were not statistically measured or adjusted.

### Conclusions

We developed and validated a model to predict multidimensional symptoms in various acute psychiatric wards using wearable sensor data and deep learning models. The constructed model proved effective in predicting a single symptom individually and multiple symptoms simultaneously. Notably, a model that predicted multiple symptoms simultaneously demonstrated more balanced within-subject classification and better between-subject symptom severity prediction. Substantial interward variations were also found in this study, suggesting that generalizability is a key issue. By discussing challenges and solutions for generalizability, we have contemplated the future direction of AI-CDSSs in acute psychiatric wards.

## Appendix

Multimedia Appendix 1 Supplementary materials.

## Abbreviations

AI: artificial intelligence
AI-CDSS: artificial intelligence–assisted clinical decision support system
AUC: area under the curve
BPRS: Brief Psychiatric Rating Scale
HAM-A: Hamilton Anxiety Scale
MADRS: Montgomery-Asberg Depression Rating Scale
MTL: multitask learning
NRMSE: normalized root mean squared error
ROC: receiver operating characteristic
SAFER: Sensor Application for Early Response in Closed Wards
t-SNE: t-distributed stochastic neighbor embedding
YMRS: Young Mania Rating Scale

## Data Availability

The datasets generated or analyzed during this study are not publicly available due to confidentiality assurances given during the informed consent process, as the study involves sensitive personal data from vulnerable participants. The analysis code is publicly available [77].
