# Correction: Comprehensive Symptom Prediction in Inpatients With Acute Psychiatric Disorders Using Wearable-Based Deep Learning Models: Development and Validation Study

**DOI:** 10.2196/69042

**Published:** 2024-12-03

**Authors:** Minseok Hong, Ri-Ra Kang, Jeong Hun Yang, Sang Jin Rhee, Hyunju Lee, Yong-gyom Kim, KangYoon Lee, HongGi Kim, Yu Sang Lee, Tak Youn, Se Hyun Kim, Yong Min Ahn

**Affiliations:** 1 Department of Neuropsychiatry Seoul National University Hospital Seoul Republic of Korea; 2 Department of Psychiatry Seoul National University College of Medicine Seoul Republic of Korea; 3 Department of IT Convergence Engineering Gachon University Seongnam-si Republic of Korea; 4 Department of Psychiatry Chungnam National University Sejong Hospital Sejong Republic of Korea; 5 Department of Computer Engineering Gachon University Seongnam-si Republic of Korea; 6 Healthconnect Co. Ltd. Seoul Republic of Korea; 7 Department of Psychiatry Yong-In Mental Hospital Yongin-si Republic of Korea; 8 Department of Psychiatry and Electroconvulsive Therapy Center Dongguk University International Hospital Goyang-si Republic of Korea; 9 Institute of Buddhism and Medicine Dongguk University Seoul Republic of Korea; 10 Institute of Human Behavioral Medicine Seoul National University Medical Research Center Seoul Republic of Korea

In “Comprehensive Symptom Prediction in Inpatients With Acute Psychiatric Disorders Using Wearable-Based Deep Learning Models: Development and Validation Study” (J Med Internet Res 2024;26:e65994), a few errors were noted.

1. The following sentence:

However, significant variations were observed across wards, which presents a key challenge…

has been corrected to:

However, significant variations were observed across wards, which present a key challenge…

2. The following sentence:

Despite lower computational costs, Multi models demonstrated equivalent or superior performance than Single models…

has been corrected to:

Despite lower computational costs, Multi models demonstrated equivalent or superior performance to Single models…

3. The following sentence:

…installed in each patient’s room and common areas

has been corrected to:

…installed in each patient’s room and common areas.

4. The following sentence:

The analysis code is publicly [77].

has been corrected to:

The analysis code is publicly available [77].

5. The following sentence:

…the YMRS performed the least accurately in in external validation…

has been revised to:

…the YMRS performed the least accurately in external validation…

The correction will appear in the online version of the paper on the JMIR Publications website on December 3, 2024, together with the publication of this correction notice. Because these corrections were made after submission to PubMed, PubMed Central, and other full-text repositories, the corrected article has also been resubmitted to those repositories.

