# Supplementary material for: Comprehensive Symptom Prediction in Inpatients With Acute Psychiatric Disorders Using Wearable-Based Deep Learning Models: Development and Validation Study
Source: J Med Internet Res. 2024 Nov 13;26:e65994. doi: 10.2196/65994 (PMC11602769; doi:10.2196/65994)
Supplement: Multimedia Appendix 1 [file jmir_v26i1e65994_app1.docx]

Multimedia Appendix 1

*Tables of contents*

# **Table S1**. Comparison of the group-level scale scores between validation sets

# **Table S2**. Comparison of the deterioration cases between validation sets

# **Figure S1**. ROC curve of the deterioration models in the HAM-A

# **Figure S2**. ROC curve of the deterioration models in the MADRS

# **Figure S2**. ROC curve of the deterioration models in the YMRS

*Abbreviations*

BPRS: Brief Psychiatric Rating Scale

HAM-A: Hamilton Anxiety Rating Scale

MADRS: Montgomery-Asberg Depression Rating Scale

YMRS: Young Mania Rating Scale

ROC: Receiver operating characteristic

# **Table S1**. Comparison of the group-level scale scores between validation sets

|  | Total | Cross-validation set | External validation set | Statistic | *P* |
| --- | --- | --- | --- | --- | --- |
| BPRS | 15.9 (9.5) | 16.0 (9.8) | 15.6 (8.4) | *t*=0.5, *df*=366.2 | .59 |
| HAM-A | 8.8 (6.0) | 8.9 (6.2) | 8.3 (5.6) | *t*=1.3, *df*=349.1 | .18 |
| MADRS | 14.1 (10.5) | 14.3 (10.9) | 13.3 (9.3) | *t*=1.3, *df*=368.1 | .21 |
| YMRS | 8.1 (7.1) | 7.8 (6.8) | 8.8 (7.7) | *t*=-1.7, *df*=291.3 | .10 |

Mean (SD) scores for BPRS, HAM-A, MADRS, and YMRS compared between cross-validation and external validation sets using Welch *t* test.

# **Table S2**. Comparison of the deterioration cases between validation sets

|  | Total | Cross-validation set | External validation set | Statistic | *P* |
| --- | --- | --- | --- | --- | --- |
| BPRS | 143 (22.9) | 107 (22.2) | 36 (25.5) | χ^2^_1_=0.5 | .47 |
| HAM-A | 163 (26.1) | 122 (25.3) | 41 (29.1) | χ^2^_1_=0.6 | .42 |
| MADRS | 180 (28.8) | 142 (29.4) | 38 (27.0) | χ^2^_1_=0.2 | .65 |
| YMRS | 176 (28.2) | 143 (29.6) | 33 (23.4) | χ^2^_1_=1.8 | .18 |

Count (%) of deterioration cases for each scale compared between cross-validation and external validation sets using chi-square test.

# **Figure S1**. Receiver operating characteristic curve of the deterioration models in the HAM-A


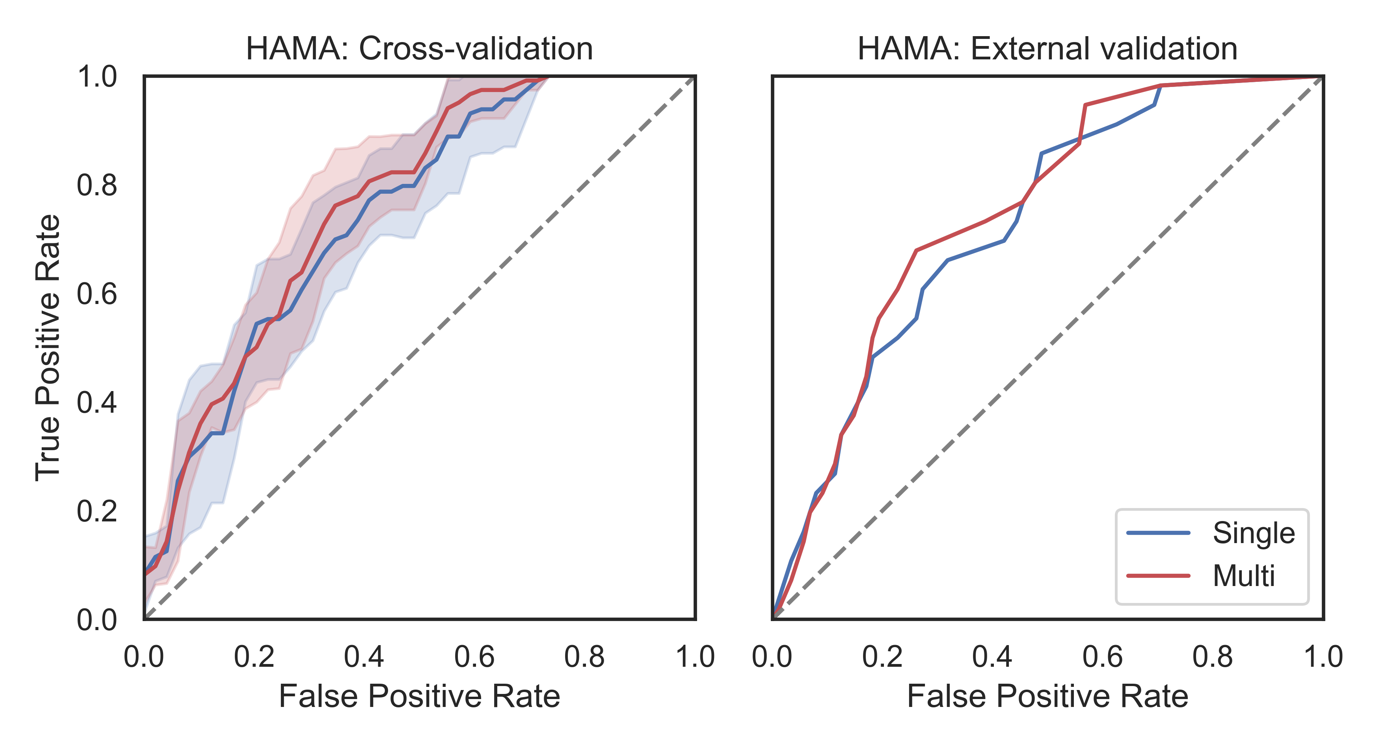


The deterioration models predicts whether HAM-A scores increased compared to the previous assessment. Colored areas on cross-validation represent the range of one standard deviation.

# **Figure S2**. Receiver operating characteristic curve of the deterioration models in the MADRS


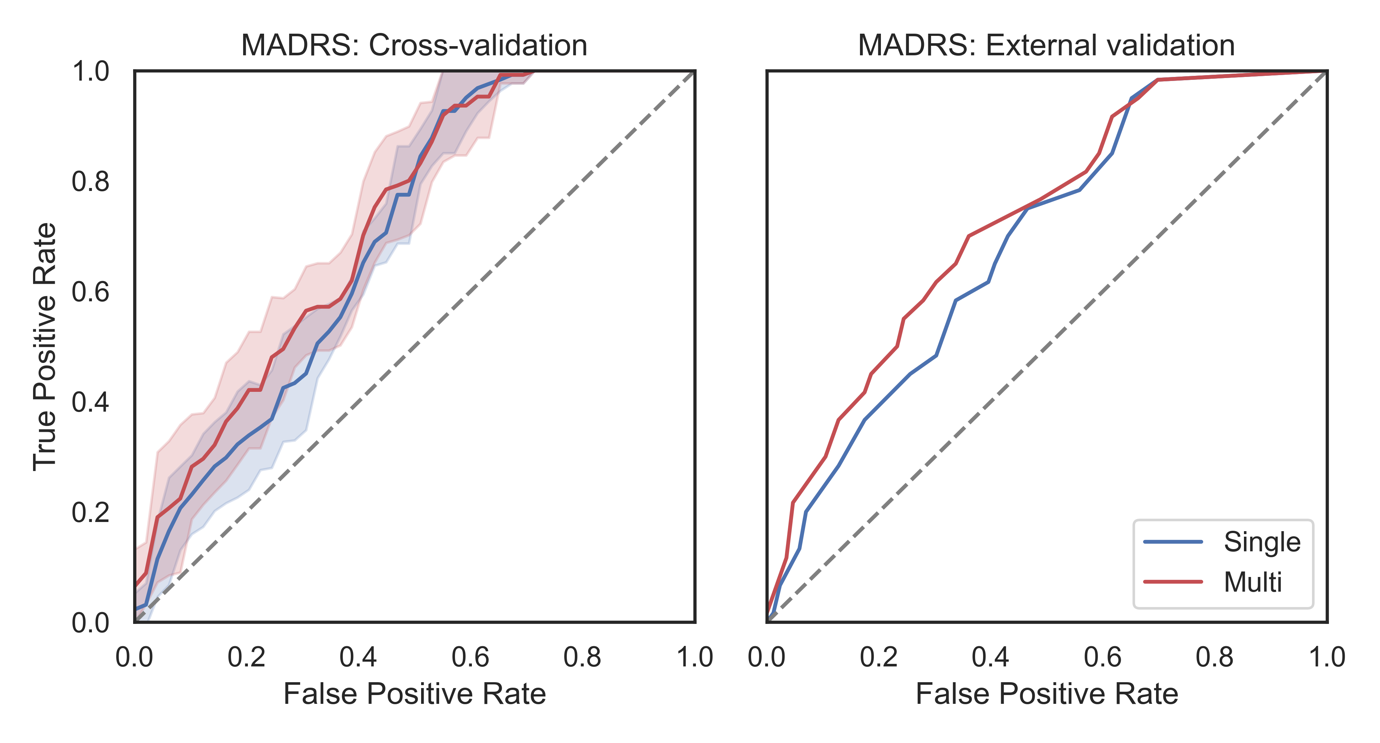


The deterioration models predicts whether MADRS scores increased compared to the previous assessment. Colored areas on cross-validation represent the range of one standard deviation.

# **Figure S3**. Receiver operating characteristic curve of the deterioration models in the YMRS


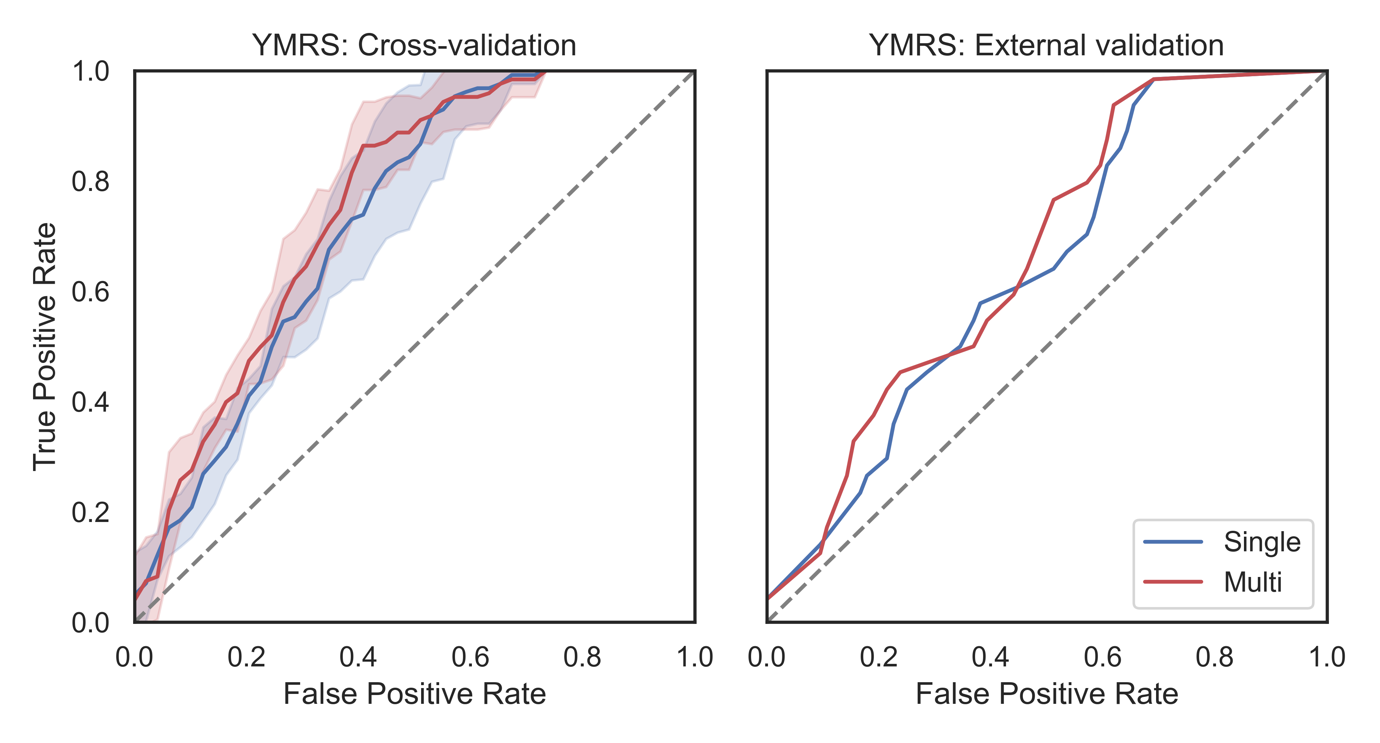


The deterioration models predicts whether YMRS scores increased compared to the previous assessment. Colored areas on cross-validation represent the range of one standard deviation.
